# Supplementary material for: A naturally arising broad and potent CD4-binding site antibody with low somatic mutation
Source: Sci Adv. 2022 Aug 12;8(31):eabp8155. doi: 10.1126/sciadv.abp8155 (PMC9374330; doi:10.1126/sciadv.abp8155)
Supplement: Supplementary file 1 — Figs. S1 to S8 Tables S1 to S6 [file sciadv.abp8155_sm.pdf]

Science Advances
AAAS

Supplementary Materials for

A naturally arising broad and potent CD4-binding site antibody with low somatic mutation

Christopher O. Barnes *et al.*

Corresponding author: Michel C. Nussenzweig, nussen@rockefeller.edu; Pamela J. Bjorkman, bjorkman@caltech.edu

Sci. Adv. **8**, eabp8155 (2022)
DOI: 10.1126/sciadv.abp8155

This PDF file includes:

Figs. S1 to S8
Tables S1 to S6

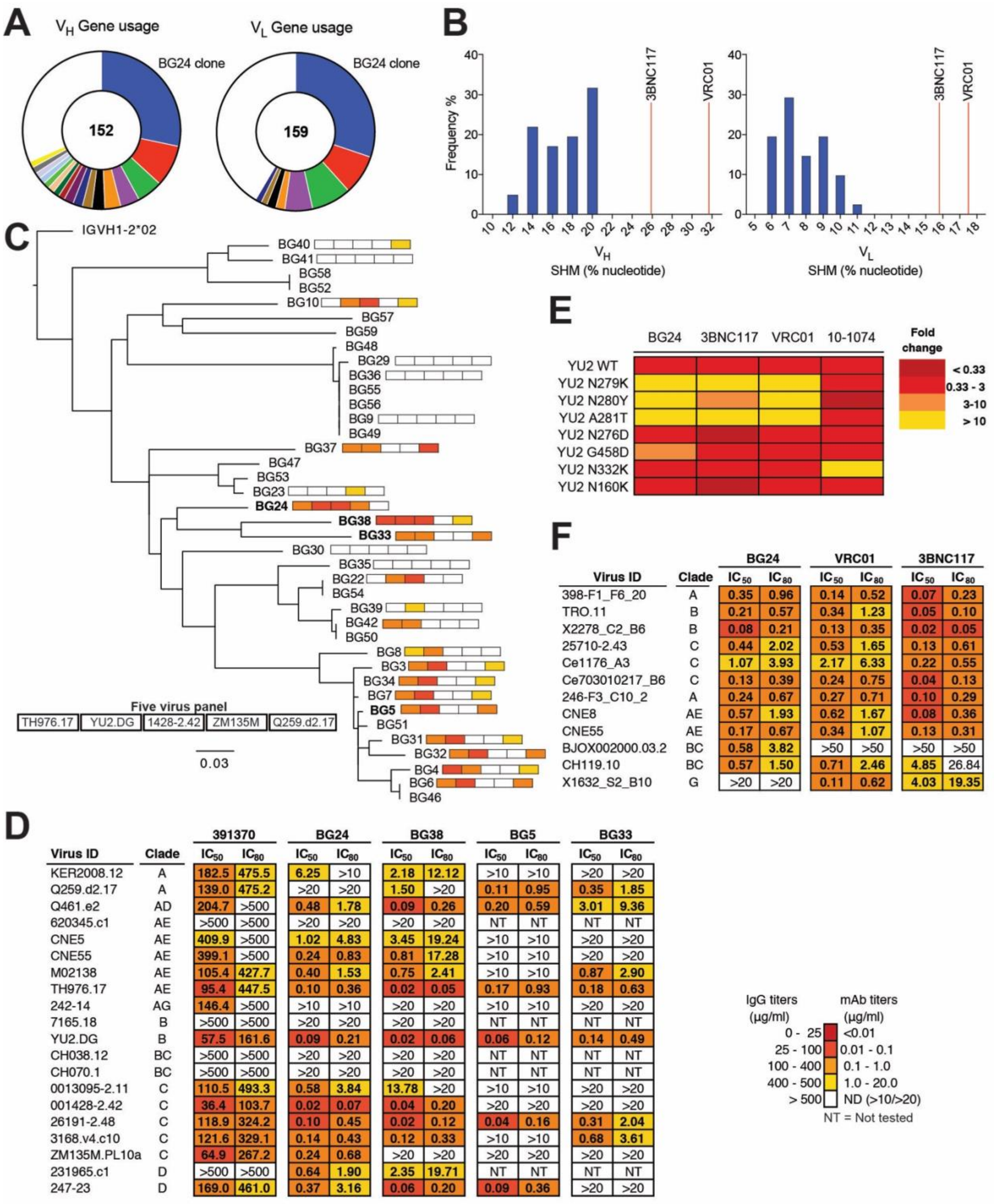

Fig. S1. Analysis of BG24 antibody family.

A) Pie charts showing the number of heavy and light chain sequences recovered from IgG$^+$ memory B-cells, with individual clones represented by slices. HC/LC members of the V_H1-

2*02/V_L2-11*01 using BG24 clone are shown as dark blue. White slices represent singlet sequences.

B) Frequency distribution of the % nucleotide somatic mutation from the putative germline variable gene in BG24 clonal family members (blue bars) for heavy chains (left) and light chains (right). Red lines denote the percentage nucleotide mutation of monoclonal antibodies 3BNC117 and VRC01.

C) Maximum-likelihood phylogenetic tree of nucleotide sequences of BG24 clone members. Colored boxes indicated mean IC_{50} neutralization values against a 5-virus panel. Names of clone members that were selected for further testing are bolded.

D) Neutralization data of BG24, BG38, BG5, BG33 and subject 391370's purified IgG against virus members of the f61 virus panel. The mean IC_{50}s in µg/mL are shown from duplicate neutralization measurements.

E) BG24, 3BNC117, VRC01, and 10-1074 neutralizing activity against a panel of HIV_{YU2} site mutant pseudoviruses. Displayed is the fold change in IC_{50} relative to neutralization of HIV_{YU2} wildtype. Data from duplicate neutralization measurements.

F) Neutralization data of BG24, 3BNC117, and VRC01 against the global 12-strain virus panel. The mean IC_{50}s in µg/mL are shown from duplicate neutralization measurements. Reference neutralization data for 3BNC117 and VRC01 sourced from the CATNAP database (*56*).

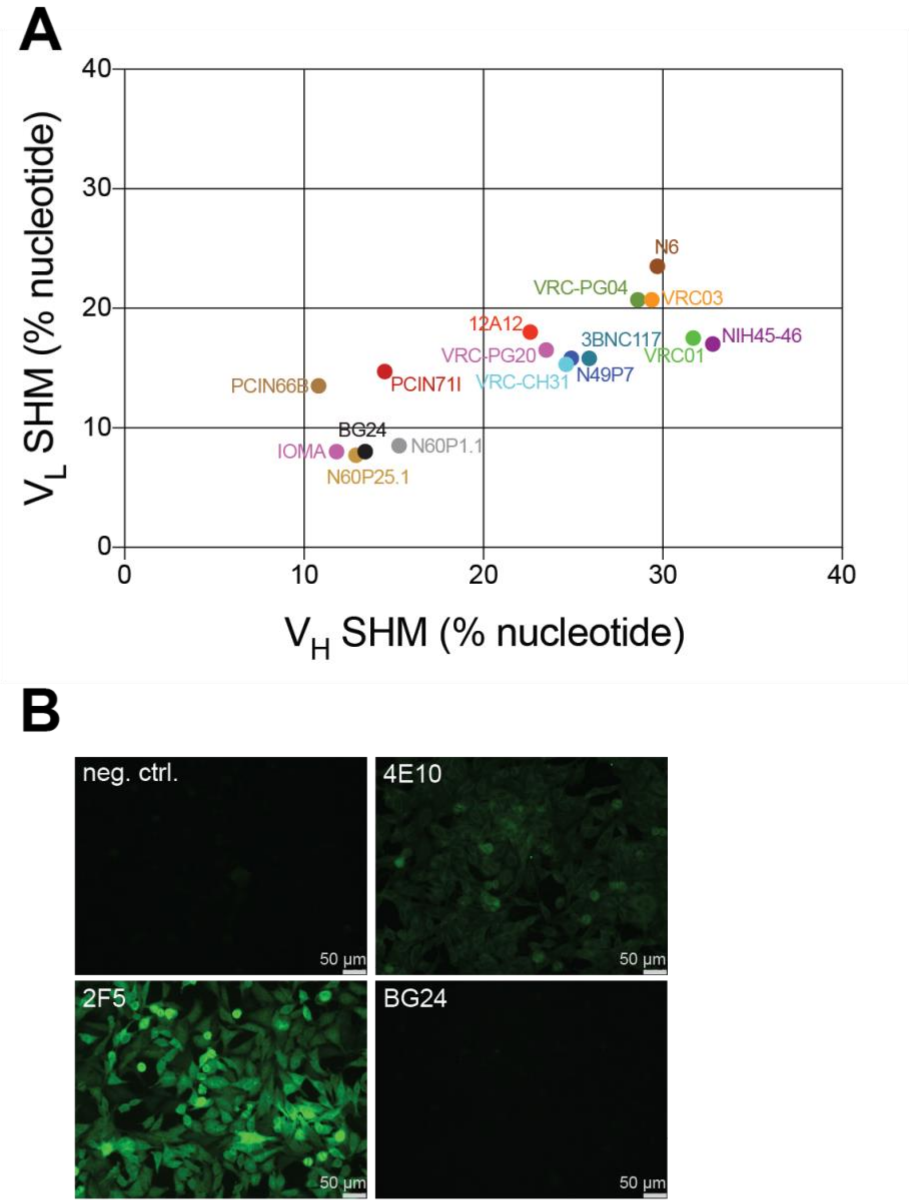

Fig. S2. Mutational analysis of BG24 family members.

A) Somatic mutation analysis of VRC01-like bNAbs, shown as % nucleotide changes relative to putative germline variable gene sequence.

B) HEp2 assay testing for potential autoreactivity of BG24 in comparison to reference antibodies 4E10 and 2F5. Testing carried out in duplicate.

A

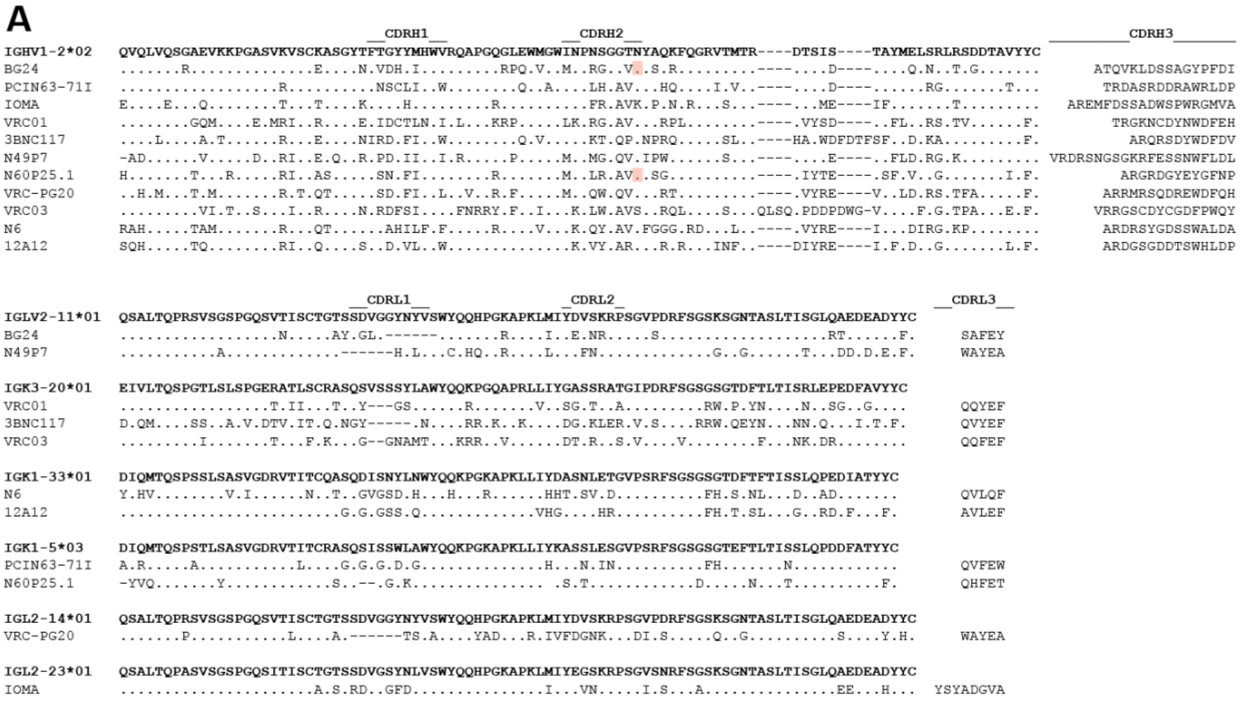

B

Virus Strain	Clade	Tier	BG24	BG24 S60A	BG24 S60T
246F3	AC	2	0.29	0.26	1.28
25710	C	1B	0.61	0.43	4.75
398F1	A	2	0.22	0.11	1.35
BJOX002000	BC	2	2.10	1.50	24.1
CE1176	C	2	1.23	0.99	10.6
TRO11	B	2	0.32	0.22	1.62
CNE8	AE	2	0.21	0.16	0.42
CE0217	C	2	0.22	0.16	0.83
CNE55	AE	2	0.60	0.37	3.69
X1632	G	2	>25	>25	>25
CH119	BC	2	0.39	0.36	2.10
X2278	B	2	0.14	0.09	0.85
BG505 T332N	A	2	0.07	0.08	0.60
		**Mean IC50**	**0.35**	**0.26**	**2.04**
		**% Breadth**	**92.3**	**92.3**	**92.3**

IC50 (µg/mL): > 25; 10 - 25; 1.0 - 10; 0.1 - 1.0; <0.1

Fig. S3. Sequence alignment of VRC01-class antibodies and N58$_{HC}$ glycan neutralization analysis of antibody BG24.

A) Sequence alignment of VRC01-class bNAbs with their putative germline V genes. CDR loops are indicated. Red boxes indicate potential N-linked glycosylation sites (PNGSs).

B) Neutralizing activity of BG24 and BG24 constructs ±N58$_{HC}$-glycan against the global 12-strain virus panel. Mean IC_{50} values from duplicate measurements are shown.

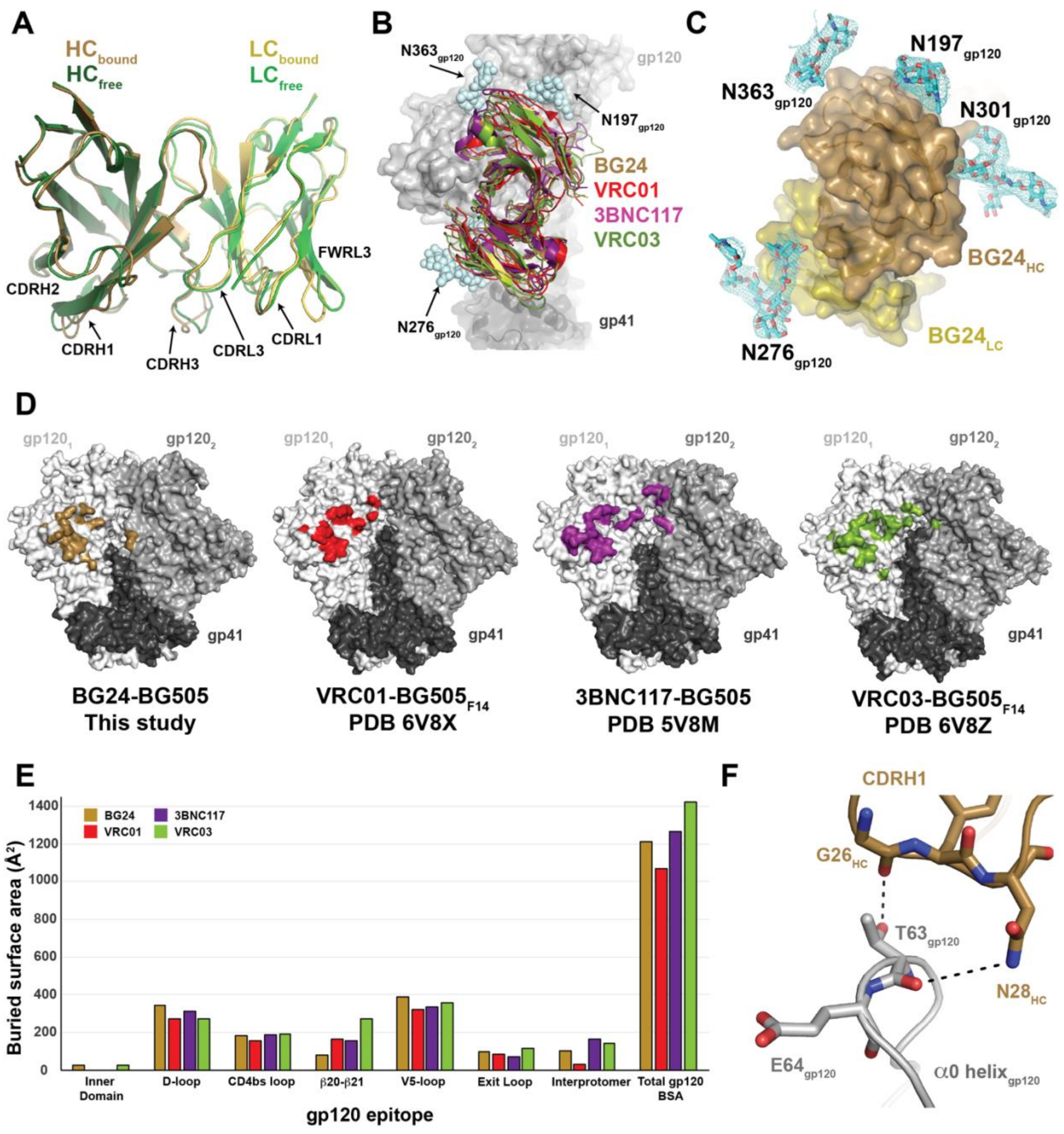

Fig. S4. Characterization of BG24 epitope on BG505 gp120.

A) Superposition of variable domains of unliganded (green) or BG505-bound (brown) BG24. CDR-loops and FWRs are indicated.

B) Superposition of variable domains of a subset of VRC01-class antibodies onto the structure of the BG24-10-1074-BG505 complex. Antibody variable domains are colored as in Figure S4D.

C) Surface representation of BG24 in the BG505-bound structure with observed gp120 CD4bs-glycans (cyan sticks). Glycan electron density (cyan mesh) contoured at 1.5σ.

D) Epitopes of CD4bs bNAbs on Env SOSIP.664 trimer structures indicated by colored surfaces on gp120 protomers. PDBs used for epitope calculations for each bNAb are shown.

E) Buried surface areas (BSA) of BG24 and VRC01-class bNAb contacts on the Env trimer surface, color-coded and derived from structures shown in panel C. BSA calculations for CD4bs regions are shown.

F) Stick representation of potential contacts between the CDRH1 of BG24 and α0 residues of the adjacent gp120 protomer of Env SOSIP.664. Black dashed lines indicate potential H-bond interactions.

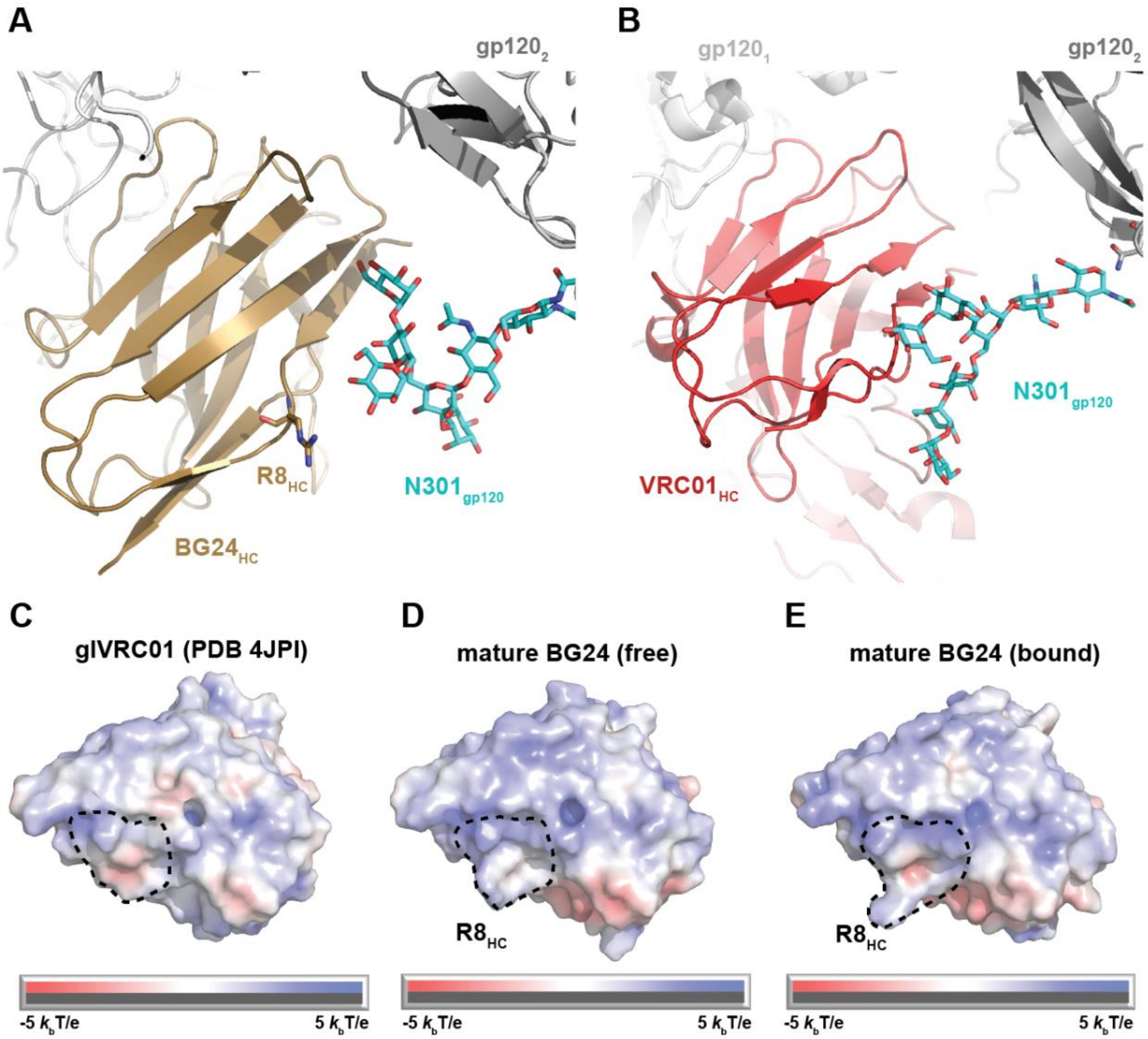

Fig. S5. Contribution of N301$_{gp120}$-glycan to BG24 maturation.

A,B) Cartoon and stick representation of potential contacts between the N301$_{gp120}$-glycan (cyan stick) from the neighboring gp120 protomer (gray) and A) BG24 (brown), or B) VRC01 (red, PDB 6VX8).

C-E) Surface representation of C) glVRC01, D) unliganded BG24, and E) BG505-bound BG24 colored by electrostatic surface potentials with red indicating negative electrostatic potential and blue representing positive electrostatic potential. Dotted line indicates potential N301$_{gp120}$-glycan footprint on antibody.

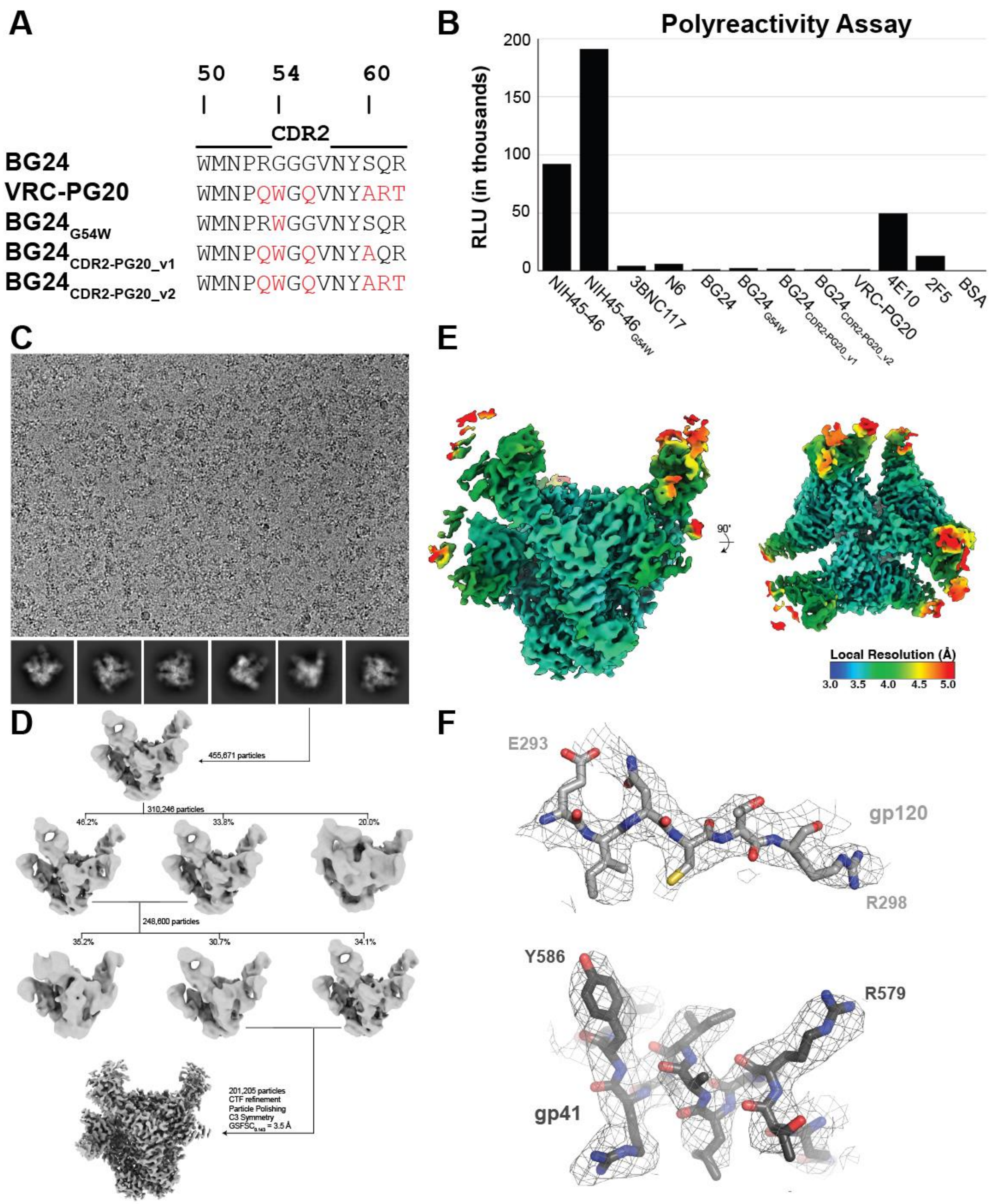

Fig. S6. Analysis of CDRH2 loop-modified BG24 variants.

A) Sequence alignment of CDRH2 amino acid residues of BG24, VRC-PG20, and engineered Phe43 pocket-filling BG24 constructs.

B) Polyreactivity ELISA-based assay detecting non-specific binding of a panel of HIV-1 bNAbs and a control protein (BSA) to a baculovirus extract.

C) Representative micrograph and 2D-class averages of $BG24_{CDR2\text{-}PG20\text{-}v2}$ – DU422 – 10-1074 complex.

D) 3D classification and refinement strategy for the $BG24_{CDR2\text{-}PG20\text{-}v2}$ – DU422 – 10-1074 complex.

E) Local resolution estimations calculated using ResMap in Relion.

F) Representative electron density contoured at 7σ for gp41 and gp120 residues (sticks).

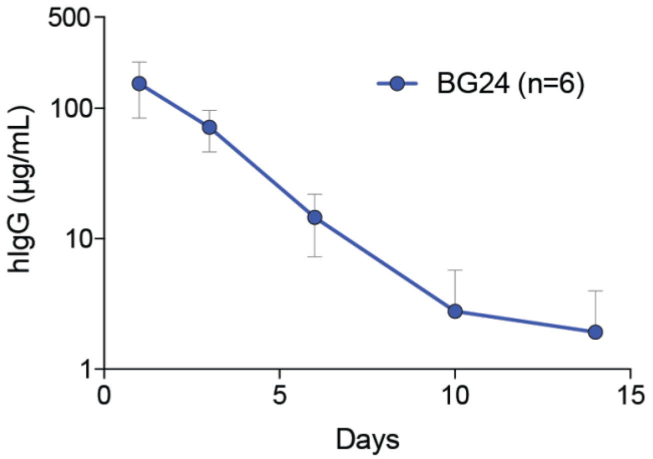

Figure S7. Pharmacokinetic analysis of BG24 in NRG mice. Analysis of BG24 concentration over time in serum of NRG mice after intravenous injection of 250 µg BG24 IgG1. Shown is the mean concentration with standard deviation of six mice from 2 independent experiments of 3 mice each.

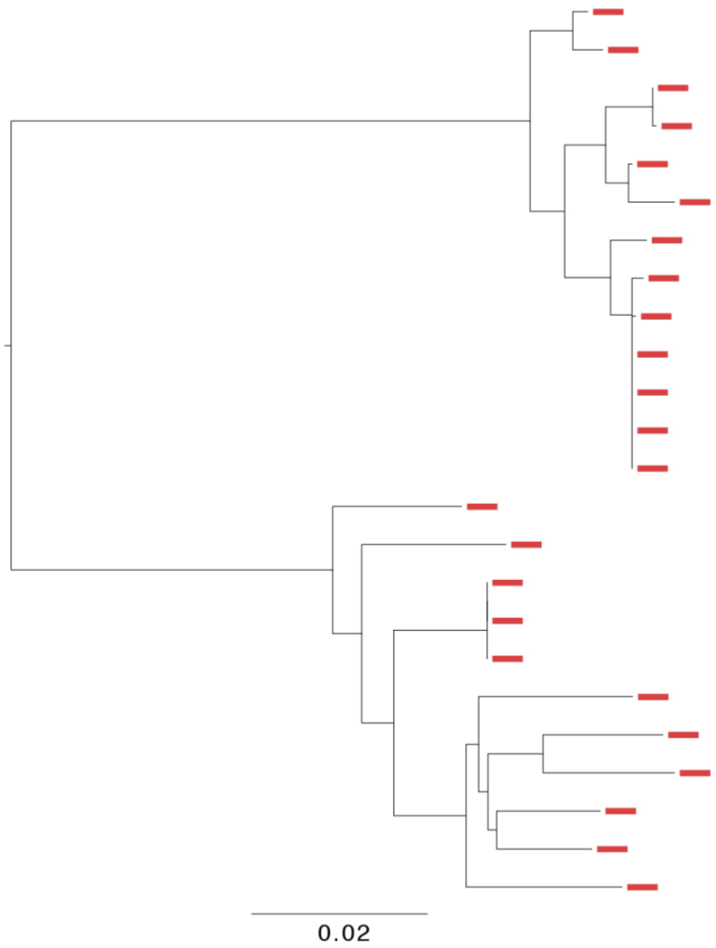

Figure S8. Phylogenetic analysis of HIV-1 env from subject 391370's plasma. Maximum-likelihood phylogenetic tree of subject 391370's plasma env nucleotide sequences. Tree is midpoint rooted.

Table S1. Plasma neutralizing activity of subject 391370 and other HIV-1 infected subjects from the HIV Controller Cohort against an early cross-clade pseudovirus panel.

Virus	Clade/Tier	Patient 3	391370	Patient 10	Patient 8	Patient 7	Patient 9
	Breadth	100%	93%	93%	93%	93%	87%
	Geomean ID80	74	132	102	97	82	186
SF162.LS	B-Tier 1A	429	2381	2381	2539	4417	4973
BaL.26	B-Tier 1B	399	854	384	460	853	680
SS1196.1	B-Tier 1B	148	242	223	88	114	304
6535.3	B-Tier 1B	21	107	33	81	177	1518
QH0692.42	B-Tier 2	56	49	89	61	27	57
SC422661.8	B-Tier 2	73	220	97	68	37	65
TRO.11	B-Tier 2	60	68	55	68	41	187
AC10.0.29	B-Tier 2	21	<20	109	34	<20	241
RHPA4259.7	B-Tier 2	104	162	96	32	87	52
THRO4156.18	B-Tier 2	40	32	26	51	38	<20
REJO4541.67	B-Tier 2	123	92	<20	312	34	23
WITO4160.33	B-Tier 2	54	102	47	31	23	156
CAAN5342.A2	B-Tier 2	36	31	70	38	73	256
PVO.4	B-Tier 3	85	113	214	134	23	25
TRJO4551.58	B-Tier 3	43	71	21	<20	46	<20
Neg. Control	MuLV	<20	<20	<20	<20	<20	<20

Breadth	ID80 titers
>90%	1000 - 10000
70-90%	250 - 1000
50-70%	50 - 250
20-50%	20 - 50
0 - 20%	<20

Table S2. Sequence analysis of BG24 family antibodies isolated from donor 391370.

	Heavy chain						Light chain					
mAb name	V-gene	% nt mutation	% AA mutation	CDRH3 (AA)	CDRH3 (seq)	Special features	V-gene	% nt mutation	% AA mutation	CDRL3 (AA)	CDRL3 (seq)	Special features
BG3	IGHV1-2*02	18.0%	31.3%	16	ATRVRLPSSDDYGFDV	6-nt insertion FWR3, 3-nt FWR1 deletion	IGLV2-11*01	7.1%	14.6%	5	SSYEF	12-nt deletion CDR1
BG4	IGHV1-2*02	20.8%	34.4%	16	ATRVKLPSSDDYGFDV	6-nt insertion FWR3, 3-nt FWR1 deletion	IGLV2-11*01	7.8%	14.6%	5	SSYEF	12-nt deletion CDR1
BG5	IGHV1-2*02	17.6%	32.3%	16	ATRVKLPSSDDYGFDV	6-nt insertion FWR3, 3-nt FWR1 deletion	IGLV2-11*01	7.1%	13.5%	5	SSYEF	12-nt deletion CDR1
BG6	IGHV1-2*02	20.1%	34.4%	16	ATRVKLPSSDDYGFDV	6-nt insertion FWR3, 3-nt FWR1 deletion	IGLV2-11*01	7.5%	14.6%	5	SSYEF	12-nt deletion CDR1
BG7	IGHV1-2*02	17.3%	31.3%	16	ATRVKLPSSDDYGFDV	6-nt insertion FWR3, 3-nt FWR1 deletion	IGLV2-11*01	7.1%	14.6%	5	SSYEF	12-nt deletion CDR1
BG8	IGHV1-2*02	19.0%	32.3%	17	ATRVKVAPSSDSYGFDV	6-nt insertion FWR3, 3-nt FWR1 deletion	IGLV2-11*01	8.6%	16.9%	5	SSYEF	12-nt deletion CDR1
BG9	IGHV1-2*02	19.0%	30.6%	16	ARQTLVTASTSFAFNN	3-nt insertion CDR1	IGLV2-11*01	5.8%	12.9%	5	SSYEY	21-nt deletion CDR1
BG10	IGHV1-2*02	16.0%	25.5%	16	ARQTLRSASASFAFNS	6-nt insertion CDR1	IGLV2-11*01	10.1%	14.1%	5	SSNEF	21-nt deletion CDR1
BG22	IGHV1-2*02	13.7%	23.7%	16	ASNVKVSSSTQYVFDV	6-nt insertion FWR3	IGLV2-11*01	9.5%	16.1%	5	SAFEF	18-nt deletion CDR1
BG23	IGHV1-2*02	11.4%	21.9%	16	ATQVKLSSSIEYPFNI	3-nt FWR1 deletion	IGLV2-11*01	7.3%	16.1%	5	SAFEF	18-nt deletion CDR1
**BG24**	**IGHV1-2*02**	**13.4%**	**22.7%**	**16**	**ATQVKLDSSAGYPFDI**	**-**	**IGLV2-11*01**	**8.0%**	**19.5%**	**5**	**SAFEY**	**18-nt deletion CDR1**
BG29	IGHV1-2*02	19.4%	30.6%	16	ARQTLVTASTSFAFNN	3-nt insertion CDR1	IGLV2-11*01	5.8%	12.9%	5	SSYEY	21-nt deletion CDR1
BG30	IGHV1-2*02	15.8%	24.7%	16	ATRVKVTSSTTFTFDI	-	IGLV2-11*01	9.2%	17.2%	5	SAFEY	18-nt deletion CDR1
BG31	IGHV1-2*02	19.0%	34.4%	16	ATRVKLPSSDDYGFDV	6-nt insertion FWR3, 3-nt FWR1 deletion	IGLV2-11*01	7.8%	15.7%	5	SSYEF	12-nt deletion CDR1
BG32	IGHV1-2*02	17.6%	30.2%	16	ATRVKLPSSDDYGFDV	6-nt insertion FWR3, 3-nt FWR1 deletion	IGLV2-11*01	7.8%	16.9%	5	SSYEF	12-nt deletion CDR1
BG33	IGHV1-2*02	14.7%	26.8%	16	ASQVAVGDSTHYPFDV	-	IGLV2-11*01	6.9%	12.8%	5	SAFEF	18-nt deletion CDR1
BG34	IGHV1-2*02	17.0%	31.3%	16	ATRVKLPSSDDYGFDV	6-nt insertion FWR3, 3-nt FWR1 deletion	IGLV2-11*01	7.1%	14.6%	5	SSYEF	12-nt deletion CDR1
BG35	IGHV1-2*02	16.2%	26.8%	16	ASNVKVPSSTVYVFDV	6-nt insertion FWR3	IGLV2-11*01	8.8%	16.1%	5	SAFEF	18-nt deletion CDR1
BG36	IGHV1-2*02	19.0%	30.6%	16	ARQTLVTASTSFAFNN	3-nt insertion CDR1	IGLV2-11*01	5.8%	12.9%	5	SSYEY	21-nt deletion CDR1
BG37	IGHV1-2*02	17.1%	25.8%	18	ATRVRLPSSSSGDYAFNI	-	IGLV2-11*01	8.8%	18.4%	5	SSYEF	18-nt deletion CDR1
BG38	IGHV1-2*02	18.2%	28.9%	16	ATQVAVAASTGWAFDV	6-nt insertion FWR3	IGLV2-11*01	10.7%	17.2%	5	SAFEY	18-nt deletion CDR1
BG39	IGHV1-2*02	14.1%	24.7%	16	ASNVKVTSSTQFVFDV	6-nt insertion FWR3	IGLV2-11*01	9.2%	16.1%	5	SAFEF	18-nt deletion CDR1
BG40	IGHV1-2*02	16.6%	32.3%	16	AVSTLMASSSTWGLNV	6-nt insertion FWR3	IGLV2-11*01	9.5%	18.4%	5	SSYEF	18-nt deletion CDR1
BG41	IGHV1-2*02	13.8%	29.2%	12	AVSTLSDFGFNI	6-nt insertion FWR3	IGLV2-11*01	9.2%	12.6%	5	SSYEF	18-nt deletion CDR1
BG42	IGHV1-2*02	13.4%	23.7%	16	ASNVKVPSSTQFVFDV	6-nt insertion FWR3	IGLV2-11*01	9.2%	17.2%	5	SAFEF	18-nt deletion CDR1
BG46	IGHV1-2*02	20.1%	34.4%	16	ATRVKLPSSDDYGFDV	6-nt insertion FWR3, 3-nt FWR1 deletion	IGLV2-11*01	7.5%	14.6%	5	SSYEF	12-nt deletion CDR1
BG47	IGHV1-2*02	13.6%	24.2%	16	ATQVKLSSSIEYPFDI	3-nt FWR1 deletion	IGLV2-11*01	6.9%	16.1%	5	SAFEF	18-nt deletion CDR1
BG48	IGHV1-2*02	19.0%	29.6%	16	ARQTLVTASTSFAFNN	3-nt insertion CDR1	IGLV2-11*01	5.8%	12.9%	5	SSYEY	21-nt deletion CDR1
BG49	IGHV1-2*02	19.0%	30.6%	16	ARQTLVTASTSFAFNN	3-nt insertion CDR1	IGLV2-11*01	5.8%	12.9%	5	SSYEY	21-nt deletion CDR1
BG50	IGHV1-2*02	13.4%	23.7%	16	ASNVKVPSSTQFVFDV	6-nt insertion FWR3	IGLV2-11*01	9.2%	17.2%	5	SAFEF	18-nt deletion CDR1
BG51	IGHV1-2*02	16.6%	30.2%	16	ATRVKLPSSDDYGFDV	6-nt insertion FWR3, 3-nt FWR1 deletion	IGLV2-11*01	7.1%	14.6%	5	SSYEF	12-nt deletion CDR1
BG52	IGHV1-2*02	16.2%	31.3%	16	AVKVLRTSSSGYGFNV	6-nt insertion FWR3	IGLV2-11*01	6.5%	12.6%	5	SSYEY	18-nt deletion CDR1
BG53	IGHV1-2*02	12.1%	24.0%	16	ATQVKLSSSIEYPFNI	3-nt FWR1 deletion	IGLV2-11*01	6.9%	14.9%	5	SAFEF	18-nt deletion CDR1
BG54	IGHV1-2*02	13.7%	23.7%	16	ASNVKVSSSTQYVFDV	6-nt insertion FWR3	IGLV2-11*01	9.5%	16.1%	5	SAFEF	18-nt deletion CDR1
BG55	IGHV1-2*02	19.0%	30.6%	16	ARQTLVTASTSFAFNN	3-nt insertion CDR1	IGLV2-11*01	5.8%	12.9%	5	SSYEY	21-nt deletion CDR1
BG56	IGHV1-2*02	19.0%	30.6%	16	ARQTLVTASTSFAFNN	3-nt insertion CDR1	IGLV2-11*01	5.8%	12.9%	5	SSYEY	21-nt deletion CDR1
BG57	IGHV1-2*02	19.0%	33.7%	16	ARQTLTTASTSFAFNH	-	IGLV2-11*01	7.0%	14.1%	5	SSYEF	21-nt deletion CDR1
BG58	IGHV1-2*02	16.2%	31.3%	16	AVKVLRTSSSGYGFNV	6-nt insertion FWR3	IGLV2-11*01	6.5%	12.6%	5	SSYEY	18-nt deletion CDR1
BG59	IGHV1-2*02	17.0%	29.6%	16	ARQTLVTASTSFAFDS	-	IGLV2-11*01	5.5%	11.9%	5	SSLEF	21-nt deletion CDR1

Table S3. 126 virus cross-clade neutralization panel data.

Virus ID	Clade*	BG24 WT IC50	BG24 WT IC80	BG24 G54W IC50	BG24 G54W IC80	BG24 CDR2-v1 IC50	BG24 CDR2-v1 IC80	BG24 CDR2-v2 IC50	BG24 CDR2-v2 IC80	BG24 Y100$_B$W IC50	BG24 Y100$_B$W IC80
6535.3	B	1.55	5.26	1.57	17.7	5.55	>25	6.58	>25	>25	>25
QH0692.42	B	0.96	2.71	0.54	1.98	0.85	3.13	0.94	3.54	0.67	2.47
SC422661.8	B	0.06	0.27	0.05	0.21	0.03	0.15	0.05	0.24	0.08	0.30
PVO.4	B	0.18	0.83	0.10	0.67	0.17	1.17	0.21	0.96	0.14	0.93
TRO.11	B	0.16	1.08	0.07	0.33	0.05	0.25	0.05	0.19	0.13	0.72
AC10.0.29	B	5.21	>25	7.27	>25	19.96	>25	11.20	>25	>25	>25
RHPA4259.7	B	0.03	0.09	0.02	0.08	0.01	0.05	0.02	0.05	0.02	0.08
THRO4156.18	B	3.01	18.7	1.20	6.04	0.98	5.33	1.20	6.38	4.48	18.0
REJO4541.67	B	0.10	0.45	0.16	1.39	0.12	0.93	0.13	0.88	>25	>25
TRJO4551.58	B	0.34	1.69	0.83	4.87	0.11	0.53	0.19	0.69	>25	>25
WITO4160.33	B	0.15	0.49	0.10	0.36	0.09	0.34	0.11	0.36	0.13	0.63
CAAN5342.A2	B	1.08	3.96	0.83	3.05	1.24	5.95	0.71	2.44	0.85	4.42
WEAU_d15_410_787	B (T/F)	0.07	0.32	0.06	0.19	0.06	0.21	0.04	0.13	0.13	0.67
1006_11_C3_1601	B (T/F)	0.08	0.33	0.07	0.24	0.09	0.30	0.12	0.39	0.09	0.33
1054_07_TC4_1499	B (T/F)	0.69	3.42	0.32	1.77	0.50	2.99	0.48	2.44	0.83	5.03
1056_10_TA11_1826	B (T/F)	0.39	1.88	0.16	1.23	0.20	1.43	0.34	1.50	0.55	2.70
1012_11_TC21_3257	B (T/F)	0.11	0.50	0.05	0.21	0.08	0.46	0.06	0.28	0.09	0.41
6240_08_TA5_4622	B (T/F)	0.41	2.82	0.35	1.62	0.38	1.75	0.37	1.67	0.54	2.60
6244_13_B5_4576	B (T/F)	0.22	0.76	0.15	0.52	0.17	0.56	0.16	0.53	0.16	0.57
62357_14_D3_4589	B (T/F)	0.38	3.74	0.13	0.96	0.09	0.40	0.17	0.77	>25	>25
SC05_8C11_2344	B (T/F)	0.79	2.63	0.61	2.05	0.70	2.44	0.50	1.39	15.47	>25
Du156.12	C	0.07	0.35	0.03	0.14	0.02	0.09	0.02	0.06	0.20	1.34
Du172.17	C	24.66	>25	0.46	5.08	0.05	0.19	0.12	1.07	>25	>25
Du422.1	C	8.38	>25	1.82	7.22	0.36	1.63	0.09	0.31	>25	>25
ZM197M.PB7	C	0.36	1.25	0.19	0.91	0.14	0.50	0.17	0.83	0.30	1.51
ZM214M.PL15	C	0.40	2.71	0.12	0.76	0.11	1.10	0.15	0.97	2.38	17.7
ZM233M.PB6	C	6.55	>25	0.67	2.92	0.44	1.87	0.21	0.68	>25	>25
ZM249M.PL1	C	0.12	0.40	0.05	0.18	0.04	0.12	0.03	0.11	0.31	2.33
ZM53M.PB12	C	0.33	1.51	0.25	0.83	0.27	1.30	0.26	1.18	1.00	6.29
ZM109F.PB4	C	0.31	2.13	0.08	0.41	0.05	0.16	0.03	0.10	>25	>25
ZM135M.PL10a	C	0.13	0.48	0.13	0.54	0.13	0.60	0.12	0.80	0.63	7.58
CAP45.2.00.G3	C	0.04	0.25	0.02	0.11	0.01	0.03	0.02	0.05	3.72	>25
CAP210.2.00.E8	C	>25	>25	6.96	>25	9.99	>25	2.43	18.72	>25	>25
HIV-001428-2.42	C	0.03	0.09	0.02	0.06	0.01	0.05	0.02	0.08	2.48	>25
HIV-0013095-2.11	C	1.29	15.4	1.28	8.92	1.69	>25	2.86	>25	>25	>25
HIV-16055-2.3	C	0.08	0.31	0.05	0.14	0.04	0.13	0.04	0.10	0.11	0.35
HIV-16845-2.22	C	3.52	24.8	1.92	8.97	1.97	15.8	1.71	8.63	4.00	>25
Ce1086_B2	C (T/F)	0.39	1.02	0.16	0.68	0.18	0.60	0.18	0.56	1.13	13.1
Ce0393_C3	C (T/F)	0.10	0.45	0.06	0.29	0.04	0.15	0.05	0.19	0.37	2.00
Ce1176_A3	C (T/F)	0.89	4.41	0.40	1.88	0.55	2.56	0.58	2.56	6.61	>25
Ce2010_F5	C (T/F)	0.25	1.24	0.12	0.56	0.17	0.78	0.31	1.06	0.25	1.14
Ce0682_E4	C (T/F)	0.05	0.26	0.03	0.08	0.02	0.08	0.04	0.15	0.05	0.21
Ce1172_H1	C (T/F)	>25	>25	>25	>25	>25	>25	>25	>25	>25	>25
Ce2060_G9	C (T/F)	0.23	1.10	0.13	0.60	0.17	0.78	0.12	0.53	0.33	2.36
Ce703010054_2A2	C (T/F)	0.69	2.47	0.17	0.83	0.07	0.32	0.09	0.40	>25	>25
BF1266.431a	C (T/F)	>25	>25	5.23	>25	8.31	>25	>25	>25	>25	>25
246F C1G	C (T/F)	>25	>25	>25	>25	4.30	>25	11.08	>25	>25	>25
249M B10	C (T/F)	0.35	1.60	0.13	0.60	0.12	0.44	0.18	0.60	1.48	8.16
ZM247v1(Rev-)	C (T/F)	0.11	0.57	0.09	0.45	0.09	0.43	0.08	0.55	0.60	3.53
7030102001E5(Rev-)	C (T/F)	2.05	7.09	1.67	5.45	0.84	2.67	0.70	2.14	>25	>25
1394C9G1(Rev-)	C (T/F)	0.53	2.49	0.17	1.12	0.25	1.78	0.22	1.00	6.44	>25
Ce704809221_1B3	C (T/F)	0.78	3.69	0.15	1.06	0.13	0.95	0.19	1.18	17.33	>25
CNE19	BC	0.06	0.28	0.04	0.17	0.04	0.21	0.04	0.22	0.06	0.36
CNE20	BC	0.47	2.45	0.19	1.02	0.19	0.92	0.32	2.72	>25	>25
CNE21	BC	0.60	2.77	0.20	1.42	0.22	0.99	0.15	0.88	>25	>25
CNE17	BC	1.00	4.88	0.59	2.76	0.79	3.60	0.96	4.69	>25	>25
CNE30	BC	0.87	2.90	0.55	1.75	0.41	1.35	0.34	1.13	2.88	16.6
CNE52	BC	0.07	0.22	0.03	0.08	0.02	0.06	0.02	0.07	0.10	0.40
CNE53	BC	0.16	0.60	0.10	0.33	0.09	0.31	0.09	0.39	0.19	0.98
CNE58	BC	0.04	0.10	0.03	0.08	0.02	0.06	0.03	0.07	0.05	0.12
MS208.A1	A	0.32	1.95	0.10	0.32	0.07	0.22	0.06	0.25	7.33	>25
Q23.17	A	0.10	0.40	0.05	0.15	0.04	0.14	0.04	0.16	0.12	0.77
Q461.e2	A	0.48	1.54	0.27	0.69	0.28	0.74	0.18	0.61	0.27	0.98
Q769.d22	A	0.09	0.36	0.05	0.17	0.05	0.16	0.06	0.19	0.09	0.33
Q259.d2.17	A	>25	>25	>25	>25	1.02	12.3	0.09	0.30	>25	>25
Q842.d12	A	0.05	0.14	0.03	0.08	0.03	0.09	0.03	0.08	0.02	0.09
0260.v5.c36	A	0.54	1.75	0.35	1.11	0.32	1.03	0.26	0.83	0.48	2.21
3415.v1.c1	A	0.29	0.93	0.12	0.38	0.08	0.27	0.06	0.20	6.65	>25
3365.v2.c2	A	0.06	0.18	0.04	0.09	0.04	0.12	0.04	0.12	0.08	0.36

Virus ID	Clade*	BG24 WT IC50	BG24 WT IC80	BG24 G54W IC50	BG24 G54W IC80	BG24 CDR2-v1 IC50	BG24 CDR2-v1 IC80	BG24 CDR2-v2 IC50	BG24 CDR2-v2 IC80	BG24 Y100$_B$W IC50	BG24 Y100$_B$W IC80
191955_A11	A (T/F)	>25	>25	>25	>25	>25	>25	11.5	>25	>25	>25
191084 B7-19	A (T/F)	0.17	0.37	0.11	0.31	0.08	0.18	0.11	0.29	0.16	0.60
9004SS_A3_4	A (T/F)	0.42	1.98	0.25	1.72	0.15	0.69	0.23	0.97	1.33	8.80
T257-31	AG	1.41	11.9	0.70	5.42	0.72	3.50	0.49	2.26	2.48	13.9
928-28	AG	0.55	1.79	0.31	1.30	0.29	0.94	0.33	1.44	1.95	8.13
263-8	AG	0.14	0.41	0.05	0.19	0.06	0.22	0.07	0.47	0.13	1.05
T250-4	AG	>25	>25	>25	>25	>25	>25	>25	>25	>25	>25
T251-18	AG	0.92	4.68	0.45	1.63	0.37	1.70	0.40	1.44	3.29	17.7
T278-50	AG	>25	>25	>25	>25	>25	>25	>25	>25	>25	>25
T255-34	AG	0.16	0.89	0.05	0.26	0.02	0.13	0.03	0.16	2.08	>25
211-9	AG	>25	>25	>25	>25	0.97	5.11	1.02	4.80	>25	>25
235-47	AG	0.40	3.30	0.07	0.34	0.03	0.10	0.03	0.10	>25	>25
620345.c01	AE	>25	>25	>25	>25	>25	>25	>25	>25	>25	>25
CNE8	AE	0.28	1.21	0.12	0.54	0.07	0.32	0.10	0.44	1.49	15.0
C1080.c03	AE	1.27	6.28	0.39	2.69	0.25	1.67	0.30	1.92	14.77	>25
R2184.c04	AE	0.07	0.29	0.02	0.10	0.03	0.11	0.03	0.14	0.07	0.29
R1166.c01	AE	0.20	0.69	0.13	0.43	0.09	0.30	0.12	0.38	0.32	1.11
R3265.c06	AE	0.51	4.70	0.06	1.18	0.17	1.22	0.14	0.86	8.17	>25
C2101.c01	AE	0.09	0.42	0.02	0.10	0.04	0.13	0.03	0.13	0.24	2.05
C3347.c11	AE	0.04	0.18	0.01	0.05	0.01	0.04	0.02	0.07	0.17	1.93
C4118.c09	AE	0.12	0.68	0.04	0.21	0.04	0.24	0.07	0.41	3.00	>25
CNE5	AE	0.96	5.22	0.18	0.80	0.11	0.48	0.11	0.49	>25	>25
BJOX009000.02.4	AE	2.52	7.22	1.56	5.49	2.04	7.11	1.54	5.34	6.57	>25
BJOX015000.11.5	AE (T/F)	0.14	1.01	0.06	0.39	0.15	0.98	0.08	0.63	0.13	1.00
BJOX010000.06.2	AE (T/F)	>25	>25	>25	>25	>25	>25	19.08	>25	>25	>25
BJOX025000.01.1	AE (T/F)	>25	>25	4.13	>25	0.04	0.23	0.12	0.71	>25	>25
BJOX028000.10.3	AE (T/F)	0.42	22.8	0.01	0.04	0.01	0.03	0.00	0.02	>25	>25
X1193_c1	G	0.29	1.34	0.08	0.27	0.04	0.19	0.08	0.32	>25	>25
P0402_c2_11	G	0.17	0.60	0.06	0.30	0.04	0.16	0.05	0.16	1.95	14.8
X1254_c3	G	0.19	0.55	0.10	0.34	0.08	0.28	0.09	0.24	3.76	>25
X2088_c9	G	>25	>25	>25	>25	>25	>25	>25	>25	>25	>25
X2131_C1_B5	G	1.05	3.29	0.19	1.56	0.08	0.50	0.12	0.76	>25	>25
P1981_C5_3	G	7.34	>25	1.63	6.44	0.48	1.67	0.44	1.51	>25	>25
X1632_S2_B10	G	>25	>25	>25	>25	0.92	>25	0.20	>25	>25	>25
3016.v5.c45	D	>25	>25	23.60	>25	2.29	20.3	1.39	16.7	>25	>25
A07412M1.vrc12	D	0.15	0.57	0.10	0.35	0.08	0.39	0.08	0.35	0.23	1.23
231965.c01	D	1.23	3.52	0.86	1.53	0.78	1.38	0.57	1.23	3.44	13.6
231966.c02	D	0.35	1.66	0.15	0.73	0.08	0.28	0.09	0.34	23.80	>25
6405.v4.c34	D	0.91	1.97	0.50	1.59	0.40	1.03	0.26	0.83	0.67	2.26
3817.v2.c59	CD	>25	>25	>25	>25	14.01	>25	21.18	>25	>25	>25
6480.v4.c25	CD	0.01	0.06	0.01	0.04	0.01	0.03	0.01	0.03	0.02	0.08
6952.v1.c20	CD	0.09	0.42	0.05	0.18	0.03	0.17	0.04	0.18	0.14	1.11
6811.v7.c18	CD	0.13	0.63	0.08	0.36	0.08	0.30	0.06	0.22	0.08	0.63
89-F1_2_25	CD	>25	>25	>25	>25	>25	>25	>25	>25	>25	>25
3301.v1.c24	AC	0.04	0.16	0.03	0.11	0.03	0.11	0.04	0.13	0.04	0.17
6041.v3.c23	AC	0.05	0.27	0.01	0.08	0.01	0.03	0.01	0.02	2.33	>25
6540.v4.c1	AC	>25	>25	>25	>25	>25	>25	>25	>25	>25	>25
6545.v4.c1	AC	>25	>25	>25	>25	>25	>25	>25	>25	>25	>25
0815.v3.c3	ACD	0.03	0.13	0.01	0.04	0.01	0.04	0.02	0.05	0.03	0.13
3103.v3.c10	ACD	1.52	4.13	0.86	2.68	0.17	0.54	0.23	0.59	>25	>25
Ce703010217_B6	C	0.13	0.66	0.07	0.25	0.05	0.25	0.11	0.39	0.19	0.73
CNE55	AE	0.24	1.09	0.11	0.51	0.05	0.22	0.10	0.45	>25	>25
246-F3_C10_2	AC	0.17	0.57	0.11	0.48	0.07	0.19	0.09	0.32	0.36	1.93
CH119.10	CRF07	0.28	1.27	0.16	0.54	0.15	0.67	0.13	0.59	0.28	1.58
BJOX002000.03.2	CRF07	0.34	1.84	0.13	0.47	0.09	0.41	0.09	0.45	8.07	>25
25710-2.43	C	0.23	1.54	0.17	0.86	0.10	0.72	0.13	0.66	0.49	3.63
AD8.MM	B	0.31	0.88	0.13	0.36	0.21	0.49	0.25	0.70	0.19	0.71
MuLV	Neg. Con.	>25	>25	>25	>25	>25	>25	>25	>25	>25	>25

IC50 (µg/mL)	
>25	(white)
1.0 - 25	(yellow)
0.1 - 1.0	(orange)
<0.1	(red)

BG24 – IC_{50}=0.29µg/mL, 84.9% breadth; $BG24_{G54W}$ – IC_{50}=0.15µg/mL, 88.1% breadth; $BG24_{PG20\text{-}CDR2\text{-}v1}$ – IC_{50}=0.15µg/mL, 92.9% breadth; $BG24_{PG20\text{-}CDR2\text{-}v2}$ – IC_{50}=0.14µg/mL, 92.1% breadth; BG24 Y100$_D$W – IC50=0.52µg/mL, 66.7% breadth

Table S4. X-ray data collection, refinement and validation statistics.

	BG24 Fab *(12-2, SSRL)*	BG24-BG505-101074 *(12-2, SSRL)*
**PDB ID**	**7UCE**	**7UCF**
**Data collection**[a]		
Space group	$C222_1$	H3
Unit cell (Å)	66.5, 120, 120	209, 209, 156
α, β, γ (°)	90, 90, 90	90, 90, 120
Wavelength (Å)	1.0	1.0
Resolution (Å)	39.28-2.0 (2.06-2.0)	39.28-3.8 (4.06-3.80)
Unique Reflections	33,354 (2,031)	24,941 (4,480)
Completeness (%)	99 (94.2)	99.6 (99.2)
Redundancy	45.1 (30.1)	11.3 (11.2)
$CC_{1/2}$ (%)	95.9 (94.2)	99.8 (21.0)
<I/σI>	11.6 (0.8)	9.7 (0.9)
Mosaicity (˚)	0.20	0.26
R_{merge} (%)	22.7 (389)	14.2 (406)
R_{pim} (%)	3.8 (77.9)	3.7 (109)
Wilson *B* -factor	33.9	188.0
**Refinement and Validation**		
Resolution (Å)	34.2-2.1	39.2-3.8
Number of atoms		
Protein	3,208	11,159
Ligand	27	792
Water	52	0
R_{work}/R_{free} (%)	20.4/24.5	25.7/27.9
R.m.s. deviations		
Bond lengths (Å)	0.004	0.0055
Bond angles (˚)	0.716	0.82
MolProbity score	2.54	1.63
Clashscore (all atom)	5.9	6.2
Poor rotamers (%)	5	0
Ramachandran plot		
Favored (%)	96	92.9
Allowed (%)	3.5	6.2
Disallowed (%)	0.5	0.9
Average *B* -factor ($Å^2$)	68.1	262.6

[a]Numbers in parentheses correspond to the highest resolution shell

Table S5. Cryo-EM data collection, refinement and validation statistics.

	$\mathbf{BG24_{CDR-v2}}$ **DU422 SOSIP.664 v4.2 10-1074**
**PDB**	**7UCG**
**EMDB**	**26443**
**Data collection and processing**	
Microscope	Talos Arctica
Camera	Gatan K3 DED
Magnification	45,000x
Voltage (kV)	200
Recording mode	counting
Dose rate (e^-/pixel/s)	13.5
Electron dose ($e^-/Å^2$)	60
Defocus range (μm)	1.0 - 2.5
Pixel size (Å)	0.869
Micrographs collected	1,989
Micrographs used	1,180
Total extracted particles	455,671
Refined particles	248,600
**Reconstruction**	
Final particles	204,220
Symmetry imposed	C3
Nominal Resolution (Å)	
FSC 0.143 (unmasked.masked)	4.1/3.5
Map sharpening *B*-factor	-110
**Refinement and Validation**	
Number of atoms	
Protein	20,391
Ligand	2,271
MapCC (global/local)	0.79/0.75
R.m.s. deviations	
Bond lengths (Å)	0.01
Bond angles (˚)	1.27
MolProbity score	1.78
Clashscore (all atom)	11.1
Poor rotamers (%)	0.1
Ramachandran plot	
Favored (%)	91.8
Allowed (%)	7.9
Disallowed (%)	0.3

Table S6. Neutralization testing of BG24 and VRC01 on an extended HIV$_{YU2}$ pseudovirus site mutant panel

			BG24 IC$_{50}$ (µg/ml)	VRC01 IC$_{50}$ (µg/ml)	BG24 Fold change relative to YU2 Wildtype	VRC01 Fold change relative to YU2 Wildtype
		YU2 Wildtype	0.05	0.06		
YU2 gp160 mutation		E102K	0.02	0.06		
	V2 Loop	N160K	0.02	0.03		
	V2 Loop	N160T	0.03	0.02		
	V2 Loop	T162I	0.03	0.05		
	V2 Loop	T162N	0.02	0.05		
	D loop	N276D	0.02	0.01		
	D loop	T278K	<0,01	0.01		
	D loop	T278A	<0,01	0.01		
	D loop	T278I	<0,01	<0.01		
	D loop	N279K	>25	>25		
	D loop	N279H	>25	0.02		
	D loop	N280Y	>25	>25		
	D loop	A281T	>25	11.95		
	D loop	T278I/A281T	>25	>25		
		N295S	0.04	0.01		
	V3 Loop	N301D	<0,01	<0.01		
	V3 Loop	N332K	0.02	0.02		
	V3 Loop	N332S	0.02	0.04		
	V3 Loop	N332Y	0.02	0.04		
	V3 Loop	S334D	0.04	0.05		
	V3 Loop	S334N	0.02	0.02		
	CD4 binding loop	G366E	0.01	<0.01		
	CD4 binding loop	N365L	0.01	<0.01		
	CD4 binding loop	I371M	0.03	0.02		
		E429K	0.04	0.06		
		N448Q	0.04	0.11		
	β23/V5 loop	G458D	0.10	0.17		
	β23/V5 loop	G459D	0.03	0.02		
	β23/V5 loop	G471R	0.04	0.04		

mAb titers (µg/ml)	Fold change
<0.01	< 0.33
0.01 - 0.1	0.33 - 3
0.1 - 1.0	3-10
1.0 - 20.0	> 10
ND (>10/>20)	
